# Personalized recommendation algorithm for rehabilitation intervention in children with autism spectrum disorder based on the cognitive diagnosis model

**DOI:** 10.3389/fpsyg.2025.1696155

**Published:** 2026-01-21

**Authors:** Tian Shu, Kanglong Peng, Qing Liu, Youqi Zhu, Jing Wang, Li Gao

**Affiliations:** 1 Jiangxi Education Evaluation And Assessment Institute, Nan Chang, China; 2 Rehabilitation Department, Shenzhen Children's Hospital, Shenzhen, China; 3 Jiangxi Provincial Education Department Teaching and Textbook Research Institute, Nan Chang, China; 4 Shenzhen Cuiyuandongxiao Middle School, Shenzhen, China

**Keywords:** ASD, cognitive diagnostic model, data-driven model, machine learning, personalized intervention

## Abstract

**Aim:**

This study applied the Cognitive Diagnostic Model (CDM) to develop a personalized recommendation algorithm for rehabilitation intervention in children and adolescents with autism spectrum disorder (ASD).

**Methods:**

A total of 3,319 children and adolescents were included. Model selections recommended the Generalized Deterministic Input, Noisy “Or” Gate Model (GDINA), to simulate the response pattern of participants in the Autism Behavior Checklist.

**Results:**

Both absolute and relative indices confirmed that the response pattern of the participants displayed acceptable fitness to GDINA. Twenty-eight symptom modalities were identified, but only 12 were assigned to over one percent of this sample. Language dysfunction is commonly observed. A diagram of the possible developmental trajectory of participants with ASD indicates that sensory and related functions can be primary targets for those with severe autistic symptoms. One possible rehabilitation route was identified in this diagram that involved 2,621 participants. A detailed personalized analysis was demonstrated in randomly selected cases from this sample.

**Conclusion:**

Our study developed a personalized recommended algorithm using CDM in designing individualized interventions for children and adolescents with ASD. First, our results confirmed the heterogeneity of ASD symptoms. Importantly, the information derived from the CDM allowed for the construction of a possible development diagram of the functions defined by ABC. Although these results are theoretically sound and reasonable, they remain data-driven. Further empirical validation, particularly through experience with rigorous design, is necessary to confirm the alignment between real-world practices and data-driven models.

## Introduction

Autism spectrum disorder (ASD) is a highly heterogeneous neurodevelopmental disorder that commonly co-exists with numerous conditions (Hodis et al., 2025; Lord et al., 2020). Recent studies have revealed that ASD is recognized as a significant global health burden (Issac et al., 2025). ASD is characterized by core disorders in social communication, restricted and repetitive behaviors (RBB), and various co-occurring symptoms (González-Cortés et al., 2019; Udhnani and Lee, 2025). These manifestations can vary widely in presentation, which contributes to the challenges of clinical diagnosis and personalized interventions (Piening et al., 2023).

Autistic symptoms are categorized into two dimensions, social communication and RBB, according to the Diagnostic and Statistical Manual of Mental Disorders, 5th Text Revision (DSM-5-TR; Mandy et al., 2012). Heterogeneity in etiology, phenotype, and outcome is a hallmark of ASD (Masi et al., 2017). Studies have found that heterogeneity in children with ASD originates not only from symptom severity but also from personal variables (e.g., age, gender, and education; Chen et al., 2025; Peng et al., 2024a). Studies have found that adolescents with ASD experience difficulties in different aspects of their daily lives; hence, specific knowledge and skills acquisition training should be tailored according to personal needs in their lives, which are not explicitly mentioned in any clinical report (Son and Nam, 2024; Tawankanjanachot et al., 2024). To date, more than 100 interventions, including medical interventions and behavioral and educational methods, have been introduced in clinical practice for ASD, but limited interventions can produce promising effects (Masi et al., 2017; Zhang et al., 2024; Zhuang et al., 2024). For now, studies have found that identifying reasonable therapeutic targets remains challenging due to the inherent heterogeneity of ASD, and current management for ASD mainly focuses on its clinical manifestation (Yenkoyan et al., 2024). One small-sample study proposed that interventions tailored based on personal traits can be more promising in reducing overall autistic symptoms compared to a one-size-fits-all protocol (Issac et al., 2025; Simione et al., 2024). This is why personalized management is needed.

Cognitive diagnosis models (CDMs) refer to a series of statistical models that attempt to simulate the relationship between observed variables (e.g., item performance) and multidimensional latent variables and produce detailed information to infer the mastery status of a set of attributes. The word “diagnosis” originates from the Greek word, which means the action of identifying the causes of problems for the purpose of classification-based decision-making. The phrase “diagnosis assessment” denotes the activities to diagnose a disorder and decide the most effective treatment protocol for the patient, or more specifically, the strengths and weaknesses in one specific content, and determine the optimal rehabilitation strategy for children with ASD in this article.

Recently, CDMs have been applied not only in personalized educational recommendations, such as English and Mathematics learning, but also in psychological and even medical science (Sideridi et al., 2022; Wu et al., 2025; Zhang and Wang, 2025). The concepts of the term “attributes” have been extended not only to academic knowledge but also to specific psychological characteristics, even diseases, and pathological traits (Templin, 2006; Torre et al., 2017; Wu et al., 2015).

In our study, researchers utilized CDMs to assess children's attributes of autistic symptoms based on their responses to the designed items. These attributes might indicate whether the performance of children reflects core symptoms or is simply due to random effects. In CDMs, these attributes are set on a binary scale where “True” or “False” of the attributes can be decided, and the attribute profiles can be served as a diagnosis report of each respondent. The fundamental logic underlying our recommendation algorithm relies mainly on hierarchical assumptions within the CDM. We assume that autism symptoms can be simulated by a series of attributes that may aggregate in some pattern (e.g., sequential order). For example, one attribute may serve as a prerequisite for mastering another attribute. The dependency between attributes forms a preliminary description of the prerequisite relationships among latent attributes, including linear, convergent, divergent, and mixed types (Leighton et al., 2004). In our study, the attribute hierarchy revealed potential developmental trajectories of autism symptoms and provided practical guidance to characterize ASD subtypes, generating personalized recommendations. Over the last century, classical test theory (CTT) and item response theory (IRT) have emerged as the dominant statistical methods in measurement research, but neither CTT nor IRT can reflect the psychological or cognitive characteristics involved in participants' responses to test items, nor can they depict participants' mastery of specific, fine-grained knowledge points (Linn, 2010). To address these limitations, CDM was utilized in our study to detect the specific structure or mechanisms of autistic symptoms and to produce detailed diagnostic information about children's behaviors. In our study, researchers utilized ABC to depict autistic symptoms in children with ASD, with the ABC test components serving as the assessment attributes, including relating, sensory, language, body use, object manipulation, and social and self-help. Then, CDM was utilized to infer children's severity of ABC components based on their responses and construct the foundation for personalized interventions.

Our research aimed to construct diagnostic references based on ABC using CDM and to answer the following questions:

1. Can the response pattern of children with ASD in ABC present a reasonable fitness to CDM?
2. Could CDM reflect symptomological differences in children with different characteristics based on ABC components or attributes?
3. Could CDM simulate progress in children with ASD according to the symptomology characteristics in our sample?
4. How to construct a personalized intervention protocol according to the results of CDA?

## Materials and methods

### Participants

This study was conducted with ethical approval from the Research and Ethics Committee. Participants were recruited from the ASD referral program provided by the maternal and childcare service center, educational institutions, and community organizations. Children who were diagnosed with ASD or who received a suspected diagnosis of ASD were appointed for multidisciplinary assessment through this program. The referred child can access the necessary intervention once a definitive diagnosis of ASD is made. A multidisciplinary team was invited to confirm the diagnosis of ASD, and the members involved were a psychiatrist with an ADOS-2 license and two senior neurologists.

Prior to administration, all subjects and/or their legal guardians signed the necessary informed consent forms. Participants were included if they were older than 2 years and fulfilled the diagnostic criteria of the DSM-5-TR version 2022, that the clinical presentation should involve three symptoms of social disorders, as well as any two manifestations of stereotyped repetitive behaviors. The detailed criteria are as follows:

Socialization disorders

1. Social-emotional interaction disorders
2. Physical motor behavioral (non-verbal communication) social disorders
3. Social relationship development disorder (development, formation, and understanding)

Stereotypical repetitive behavior

1. Repetition of stereotyped motor movements, object manipulation, or verbal expressions
2. Development of repetitive, routine, and patterned stereotyped verbal or non-verbal behaviors
3. Extremely limited, fixed interests, or attention spans
4. Abnormal responses (extreme sensitivity or the opposite) to sensory input, both normal and abnormal (environmental)

For specific diagnostic criteria, refer to the suggested judgment criteria for each entry by Rice et al. (2022).

Children with other unrelated conditions were excluded from the study.

### Measure

#### Children's autism rating scale first edition

The Children's Autism Rating Scale First Edition (CARS1) was constructed to gather information about autistic symptoms from interviews with caregivers and clinical observations. CARS1 contained 15 items, including relation to people, imitation, emotional response, body use, object use, adaptation to change, visual response, listening response, sensory response, emotion, verbal communication, gesture, activity status, intellectual response, and overall impressions. A four-point rating scale is used to rate symptom severity, where one point denotes normal behavior and four points denote problematic behaviors that are different from typical developmental peers. In our study, CARS1 was delivered by trained or licensed clinicians or researchers to categorize children with ASD into three severity levels: non-autism, mild to moderate autism, and severe autism.

#### Autism behavior checklist (ABC)

The ABC possibly involves all typical autistic behaviors in children with ASD, and items are categorized into five components: sensory, relating, body use and object manipulation, language, and social and self-help. Each item was assigned different points from one to four according to its contribution to the ASD diagnosis (Krug et al., 1980). The interrater reliability is 0.85, and the intra-rater reliability is 0.82 (Abdelmageed et al., 2024; Krug et al., 1980). The cut-off score was set at 68 points to differentiate between non-autism and autism and 67 points to indicate severe autistic symptoms (Wadden et al., 1991).

### Data analysis

#### Attribute

In our study, autistic symptom attributes were defined as the necessary components for measuring ASD symptoms. ABC measures ASD symptoms in five areas: relating, sensory, language, body use, object manipulation, and social and self-help. Table 1 depicts the five attributes in ABC with detailed definitions organized based on the DSM-5-TR.

**Table 1:** Attribute definitions of ABC.

**Code**	**Attribute**	**Content definition**
S	Sensory	Sensory dysfunction is defined by atypical responses to sensory input or unusual interests in sensory aspects of the environment including: hyper- or hypo-reactivity to sensory input (e.g., apparent indifference to pain/temperature, adverse responses to specific sounds or textures, excessive smelling or touching of objects), unusual interest in sensory aspects of the environment (e.g., fascination with lights or spinning objects, seeking out specific sensory experiences)
R	Relating	Relating dysfunction is defined by persistent deficits in developing, maintaining, and understanding relationships, including difficulties adjusting behavior to suit various social contexts (e.g., understanding how to act in a job interview vs. a casual setting), challenges in sharing imaginative play or making friends, often accompanied by a lack of interest in peers or atypical social approaches (e.g., aggressive or disruptive behavior), absence of typical social reciprocity, such as one-sided friendships or relationships based solely on shared special interests .
B	Body use and object manipulation	Body use and object manipulation dysfunction is defined by the restricted, repetitive patterns of behavior, interests, or activities (e.g., hand-flapping, finger-twisting, whole-body rocking, spinning, lining up toys, spinning wheels of cars, flicking switches) that are abnormal in intensity, frequency, or form relative to age and cultural expectations. These behaviors constitute persistent motor or object-related routines that serve no obvious instrumental purpose and are not better explained by other developmental or neurological conditions
L	Language	Language dysfunction is defined by deficits in non-verbal communicative behaviors used for social interaction and social-emotional reciprocity including atypical production or use of spoken language, marked impairment in the pragmatic use of language, difficulty integrating verbal communication with non-verbal communicative behaviors such as eye contact, facial expression, and gesture, failure to initiate or sustain conversational exchanges that are appropriate to developmental level and cultural expectations
SS	Social and self-help	Social and self-help dysfunction is defined as the persistent impairment in age-appropriate social functioning combined with clinically significant delays or atypical patterns in development of self-care and other adaptive skills necessary for daily living

#### Q-matrix

Table 2 presents the Q-matrix, which is used to present how test items examine specific attributes, where zero indicates that the attribute is not examined, and one indicates that the attribute is examined. In this study, ABC items were selected as evaluation items. To confirm the Q-matrix, two senior rehabilitation experts reviewed the 57 items in the Q-matrix and confirmed their relationships with the attributes. Any conflicts were resolved by another psychologist with ADOS-2 certification.

**Table 2:** Q-matrix of ABC.

**Item code**	S^*^	R^*^	B^*^	L^*^	SS^*^
Item1	0	0	1	0	0
Item2	0	0	0	0	1
Item3	1	0	0	0	0
Item4	0	0	0	1	0
Item5	0	0	1	0	0
Item6	1	0	0	0	0
Item7	0	1	0	0	0
Item8	0	0	0	1	0
Item9	0	0	1	0	0
Item10	1	0	0	0	0
Item11	0	0	0	1	0
Item12	0	0	1	0	0
Item13	0	1	0	0	0
Item14	0	0	0	0	1
Item15	0	0	0	1	0
Item16	0	0	1	0	0
Item17	0	1	0	0	0
Item18	0	0	0	1	0
Item19	0	0	0	0	1
Item20	0	0	0	1	0
Item21	1	0	0	0	0
Item22	0	0	1	0	0
Item23	0	0	0	0	1
Item24	0	1	0	0	0
Item25	0	1	0	0	0
Item26	1	0	0	0	0
Item27	0	1	0	0	0
Item28	0	1	0	0	0
Item29	0	0	0	1	0
Item30	0	0	1	0	0
Item31	0	0	0	0	1
Item32	0	0	0	1	0
Item33	0	1	0	0	0
Item34	1	0	0	0	0
Item35	0	0	1	0	0
Item36	0	0	0	0	1
Item37	0	0	0	1	0
Item38	0	1	0	0	0
Item39	1	0	0	0	0
Item40	0	0	1	0	0
Item41	0	0	0	0	1
Item42	0	0	0	1	0
Item43	0	1	0	0	0
Item44	1	0	0	0	0
Item45	0	0	0	0	1
Item46	0	0	0	1	0
Item47	0	1	0	0	0
Item48	0	0	0	1	0
Item49	0	0	0	0	1
Item50	0	0	0	0	1
Item51	0	0	1	0	0
Item52	1	0	0	0	0
Item53	0	0	1	0	0
Item54	0	0	1	0	0
Item55	0	0	0	0	1
Item56	0	0	0	1	0
Item57	1	0	0	0	0

R, relating; S, sensory; L, language; B, body use and object manipulation; SS, social and self-help.

#### Model selection

Initially, we attempted to choose the most optimal CDM among the commonly used models with different cognitive assessment assumptions. To determine the optimal model, we selected eight models that are commonly used in clinical practice, and the true model was decided by comparison (Table 3). The evaluation process is conducted using R.

**Table 3:** Diagnosis models used in model selection.

**Model**	**Description**	**Kernel rule**
DINA, Deterministic Input, Noisy “And” gate	A correct response is only probable when every required attribute is mastered	“All-or-none” mastery demanded
DINO, Deterministic Input, Noisy “Or” gate	Mastery of at least one required attribute is sufficient for a high probability of success	“Any one will do”
ACDM, Additive Cognitive Diagnosis Model	The probability of success increases additively with the weighted sum of mastered attributes	“More skills indicate higher score”
GDINA, Generalized DINA	A saturated log-linear model that nests DINA, DINO, and all possible conjunctive/disjunctive interactions	“One model fits all rules”
LCDM, Log-linear Cognitive Diagnosis Model	A re-parameterization of GDINA that expresses any CDM as a parsimonious log-linear equation	“Lego bricks for CDMs”
LLM, Linear Logistic Model	A further constrained version of LCDM with only main-effect terms, yielding the simplest compensatory structure	“Ultra-light additive form”
RRUM, Reduced Reparameterized Unified Model	A middle-ground compensatory model that retains a reduced set of interaction terms	“Compensatory but lean”
Mixed Model	An estimation framework in which different items can follow different CDMs within the same assessment	“Mix-and-match per item”

## Result

### Demographic data

Our study collected a sample of 3,319 children and adolescents. Table 4 presents the demographic data. Our sample mainly represents the population aged around 44.77 ± 23.52 months. Most of the sample comprised men (e.g., male/female = 2,645/674). Our study tried to recruit a sample with a reasonable proportion of children and adolescents based on the Chinese Education System (e.g., nursery, 0–3 years; kindergarten, 3–6 years; primary school, 6–12 years; junior high school, 12–15 years; and high school, 15–18 years), but we only obtained a sample of 1,414 children in nursery, 1,503 children attending kindergarten, 380 registered in primary school, 19 children and adolescents from junior high school, and three from high school. For autistic symptom severity, we recruited a sample with a balance in the CARS1 classification references.

**Table 4:** Participant demographic data.

**Variables**	**Mean (SD)/Count (%)**
Sample	3,319
**Gender**	
Male	2,645 (79.69%)
Female	674 (20.31%)
**Age (months)**	
Overall	44.77(23.52)
0–35/infant	1,414 (42.60%)
36–71/kindergarten	1,503 (45.28%)
72–143/primary	380 (11.45%)
144–180/junior high	19 (0.6%)
179–216/high_school	3 (0.07%)
**ASD symptom (CARS1 classification)**	
**Symptom level**	
Non-autism	1,135 (34.20%)
Mild to moderate autism	1,446 (43.57%)
Severe Autism	738 (22.23%)

S.D., standard deviation; CARS, children autism rating scale.

### Model comparison

In our study, model comparisons were conducted among the DINA, DINO, ACDM, G-DINA, LCDM, LLM, RRUM, and mixed models. One dimensionality was confirmed in ABC in our previous work; therefore, mixed models may not work for our current data (Von Davier, 2010; Peng et al., 2024a). Therefore, detailed comparisons were conducted among DINA, DINO, ACDM, G-DINA, LCDM, LLM, and RRUM. Table 5 shows that GDINA had the smallest AIC values. We chose RMSEA2 as the absolute index to evaluate the model fitness because RMSEA2 is smaller than 0.089.

**Table 5:** Parameter statistics of different models.

**Model**	**DINA**	**DINO**	**ACDM**	**GDINA**	**LCDM**	**LLM**	**RRUM**
Npars	145	145	145	145	130	145	145
RMSEA2	0.0726	0.073	0.073	0.073	0.073	0.073	0.073
Deviance	151,162.42	151,162.42	151,162.43	151,162.42	151,196.7618	151,162.42	151,162.75
AIC	151,452.42	151,452.42	151,452.43	151,452.42	151,456.7618	151,452.42	151,452.75
BIC	152,337.99	152,337.99	152,338	152,337.99	152,250.7262	152,338	152,338.33

In summary, our data present the optimal fitness for GDINA.

GDINA is built based on DINA (deterministic inputs, noisy “and” gate model, DINA); hence, our study introduces DINA before GDINA.

DINA is written as follows:


$$\begin{array}{l} P\left(x_{ij}=1|\alpha_{i}\right)={\left(1-s_{j}\right)}^{\eta_{ij}}{g_{j}}^{1-\eta_{ij}} \end{array}$$

In the abovementioned formula, *i* denotes participants, and *j* denotes testing items. In our study, *x*_*ij*_ denotes the responses of the participants *i* to items *j*. *x*_*ij*_ is set as a binary variable that is equal to either 1 or 0. Here, 1 denotes that behaviors are observed, and 0 denotes that behaviors are absent.

All the conditions are defined by two parameters *g*_*ij*_ and *s*_*ij*_ (e.g., one item selected from the language dysfunction attribute is denoted by *a*, and 1 denotes dysfunctions are confirmed, and vice versa):

Behavior observed in children without language disorders:


$$\begin{array}{l} g_{ij}=P\left(x_{ij}=1|a=0\right) \end{array}$$

Behavior is absent in children without language disorders:


$$\begin{array}{l} {1-g}_{ij}=P\left(x_{ij}=0|a=0\right) \end{array}$$

Behavior is absent in children with language disorders:


$$\begin{array}{l} s_{ij}=P\left(x_{ij}=0|a=1\right) \end{array}$$

Behavior is observed in children with language disorders:


$$\begin{array}{l} 1-s_{ij}=P\left(x_{ij}=1|a=1\right) \end{array}$$

In this study, the symptom modality is denoted by a vector *q*_*jk*_, and the function status is denoted by *a*_*jk*_. We used η_*jk*_ to denote the ideal response of the participant *i* on item *j*, the formula is written as follows:


$$\begin{array}{l} \eta_{ij}=\Pi_{k=1}^{K}{\alpha_{ik}}^{q_{jk}} \end{array}$$

The capital *K* denotes the number of dysfunctions that *K* was set to 5, and *k* denotes dysfunctions (e.g., *k* is language). If dysfunction is confirmed is in *i*, then *a*_*jk*_ is equal to 1, and vice versa. *q*_*jk*_ denotes vectors that the interaction between items *j* and dysfunctions. For example, if item *j* reflects dysfunction *k* (e.g., *k* is language), then *q*_*jk*_ is equal to 1.

In our study, DINA assumes that, when *i* displays the dysfunction reflected by the item *j*, η_*ij*_ is equal to 1, and vice versa.

GDINA can be written as follows based on DINA:


$$\begin{array}{l} P\left(\alpha_{lj}^{*}\right)=\delta_{j0}+\overset{K_{j}^{*}}{\underset{k=1}{\sum }}\delta_{jk}\alpha_{ljk}+\overset{K_{j}^{*}}{\underset{k^{\prime }=k+1}{\sum }}\overset{K_{j}^{*}-1}{\underset{k=1}{\sum }}\delta_{jkk^{\prime }}\alpha_{ljk}\alpha_{ljk^{\prime }}\\ \;+\cdots {+\delta }_{j12\cdots K_{j}^{*}}\overset{K_{j}^{*}}{\underset{k=\;1}{\prod }}\alpha_{ljk}. \end{array}$$

GDINA utilizes δ to emphasize the interactions between different dysfunctions. This means that GINA would consider, for example, if language and sensory dysfunctions are confirmed simultaneously, what consequence would be brought about by the interaction between these two dysfunctions compared to either language or sensory dysfunction being confirmed.

### Symptom modality

Twenty-eight symptom modalities were identified in our sample, and Table 6 displays twelve symptom modalities that were assigned to more than one percent of our sample. Table 6 shows that the pattern coded in 1 (e.g., “00000”) was assigned to 1,053 (31.7%) participants, which is the predominant symptom pattern. This means that these children and adolescents did not present true autistic symptoms; instead, those behaviors may be observed at random. The pattern coded as 2 (e.g., “11111”) was assigned to 793 participants (23.9%) and ranked secondary to pattern 1. Parametric and non-parametric correlation analyses have proved that the number of dysfunctions is related to the total score (e.g., Pearson correlation index, 0.755, 0.00; Spearman index, 0.777, 0.00).

**Table 6:** Symptom modalities identified by GDINA.

**Pattern code**	**Binary code**	**Sample**	**Proportion**	**Accumulated proportion**	**Attributes mastered**
1	00000	1,053	31.7	31.7	0
2	11111	793	23.9	55.6	5
3	00011	271	8.2	63.8	2
4	00010	228	6.9	70.7	1
5	11011	222	6.7	77.3	4
6	00111	215	6.5	83.8	3
7	11000	140	4.2	88	2
8	11100	76	2.3	90.3	3
9	11001	66	2	92.3	3
10	11101	59	1.8	94.1	4
11	00100	51	1.5	95.6	1
12	00101	43	1.3	96.9	2

### Attribute mastery

In our study, attribute mastery denotes that the dysfunctions defined by these attributes are confirmed in children and adolescents. This means that children and adolescents with confirmed dysfunctions are more likely to exhibit behaviors assigned to these dysfunctions. Hence, this study used probability to describe attribute mastery in our sample.

Our study found that language dysfunction (53.6%) ranked first in terms of the probability of confirmed dysfunction. This finding indicates that language dysfunction is more likely to be observed in children and adolescents with ASD. Social and self-help (51.5%) were ranked secondary to language. The attributes ranked after social and self-help were related (43.1%), sensory (41.8%), and body use and object manipulation (38.4%).

An intervention diagram was constructed based on cluster analysis.

Our study established a recommended intervention diagram. First, symptoms confirm the probability retrieved from the CDA. The symptom confirmed probability was calculated based on the expected a posteriori method (EAP). To define, confirm, or reject, the probability cut-off value was set at 0.5. This means that confirmation is defined by a probability larger than 0.5, and vice versa. Second, cluster analysis was conducted using the Ward linkage method, or the minimum variance method, to detect potential clusters in our sample. To determine the optimal cluster size, we used the classical elbow method based on Bayesian and Akaike information criteria.

**Figure 1:**
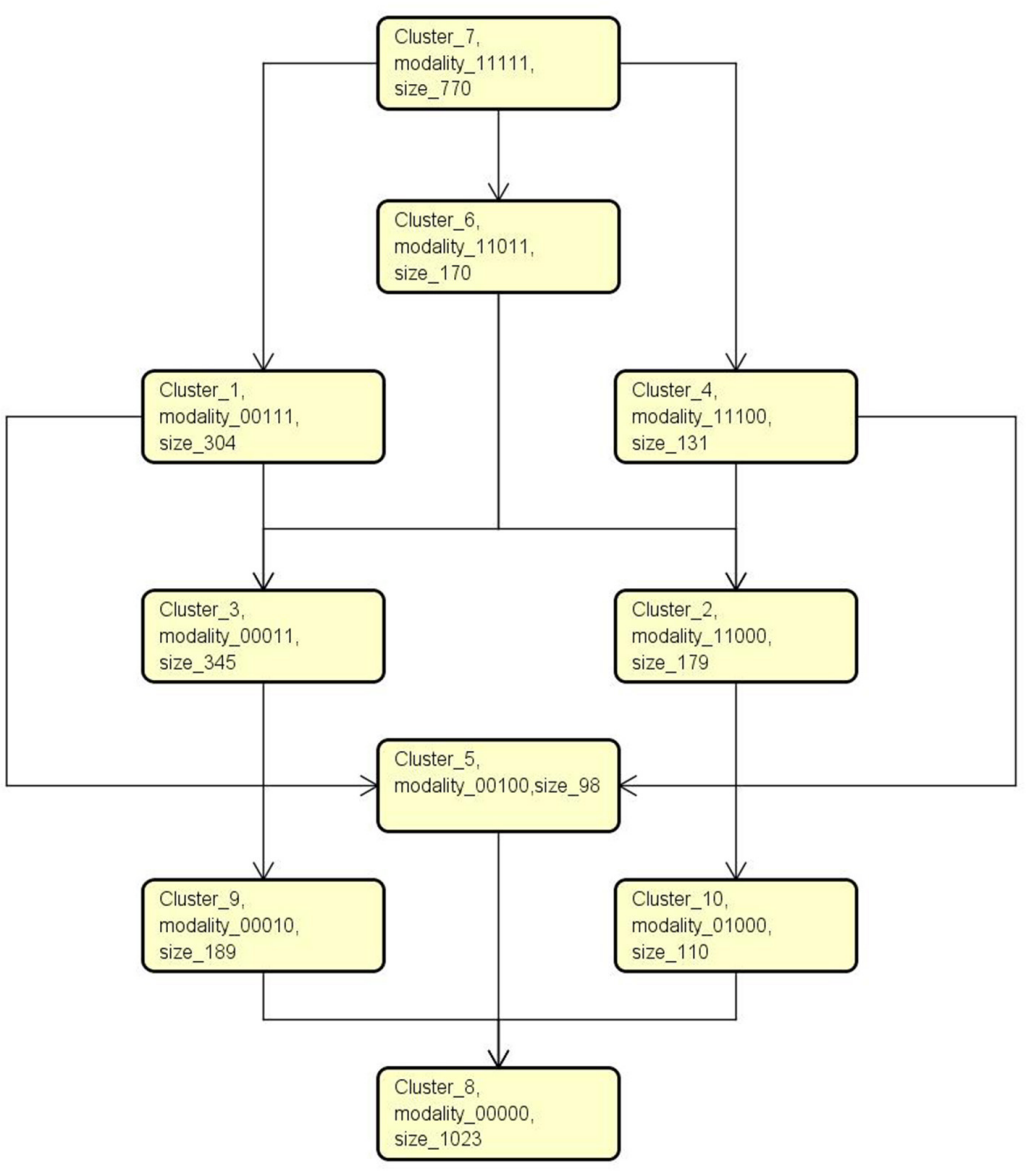
An Intervention diagram constructed based on cluster analysis.

Figure 1 shows six types of development diagrams for children and adolescents found in our study. This means that children and adolescents with ASD can learn to restore or compensate for their dysfunction by following at least six possible diagrams. Among them, the most common guideline was the one formed by clusters 7, 1, 3, 9, 8, and 2,621 (2,621/3,319, 78%), and participants were involved in this trajectory. This means that sensory and related functions can be set as prior intervention targets in those with typical autistic symptoms. Body use, object manipulation, and social and self-help are secondary to them. Language is focused on the last stage. This diagram was established to provide a possible prognosis for children and adolescents with different symptom modalities.

### Personalized analysis in clinical practice

In clinical practice, the overall assessment score is commonly used to evaluate patients based on CCT. In our recent studies, we confirmed that item and person scores (e.g., how an item can distinguish a person and how a person responds to items) can make amendments to current assessment interpretations (Peng et al., 2024a,b). However, the interactions between participants and attributes were assumed to follow one pattern. For example, in one of our studies focusing on ABC, we assumed that children with severe autistic symptoms would display more behaviors (Peng et al., 2024a). Hence, a one-size-fits-all intervention would be delivered to those who score the same points in the current clinical practice. Our study provides examples under two conditions to illustrate how CDM can be applied to real-world scenarios.

### Different symptom modalities in children and adolescents with the same scores

Two participants were randomly selected from those who scored 31 points on CARS1 and 70 points on ABC. Table 7 shows that although they scored the same in CARS1 and ABC, two different symptom modalities were recognized. ID 1395 displays the symptom modality coded by 10 (e.g., 11101), which means that language dysfunction is not confirmed in ID 1395. ID1423 presented the second predominant pattern, coded by 2 (e.g., 11111). A significant difference was found in the probability of confirming language dysfunction (e.g., 1.1% for ID1395 and 100% for ID1423). Slight differences were observed in sensory (e.g., 100% for ID1395 and 99.9% for ID1423), body use and object manipulation (e.g., 92.7% for ID1395 and 98.9% for ID1423), and social and self-help (e.g., 88% for ID1395 and 99.8% for ID1423).

**Table 7:** Cognitive diagnosis Analysis results of two children with same CARS and ABC performance.

**ID**	**Gender**	**Age/months**	**CARS**	**ABC**	**gender**	**Education**	**Symptom modality**	**S**	**R**	**B**	**L**	**SS**
ID1395	Male	50	31	70	Male	Kindergarten	11101	1	1	0.927	0.011	0.88
ID1423	Male	44	31	70	Male	Kindergarten	11111	0.999	1	0.989	1	0.998

These examples indicate that although children and adolescents may score exactly the same points in clinical assessment, they would process different attribute mastery patterns. In such cases, it is not reasonable to deliver the same intervention protocol for ID1395 and ID1423.

### Different response patterns in children and adolescents with the same symptom modality

Our study also found that although children and adolescents may display the same symptom modality, they may perform differently due to different probabilities of confirming dysfunctions. We randomly selected four examples from those who displayed a symptom modality coded as 8 (e.g., 11100; Table 8). We found that these four examples did not present language dysfunction. Although sensory, relating, body use, and object manipulation dysfunctions were confirmed in these participants, none performed the same way as the others. This can be explained by the fact that the probability of confirming these dysfunctions was different in these four participants.

**Table 8:** Different responses pattern in children and adolescents with the same symptom modality.

**Symptom modality**	**ID**	**Gender**	**Age/months**	**Eductation**	**Cars_Total _Score**	**Total score**	**Gender**	**S**	**R**	**B**	**L**	**SS**
11100	ID1945	Male	42	Kindergarten	33	33	Male	0.645	0.857	0.683	0	0.042
11100	ID643	Male	52	Kindergarten	35	37	Male	0.903	0.819	0.683	0	0.009
11100	ID1579	Male	82	Primary school	35	40	Male	0.557	0.579	0.501	0	0.252
11100	ID2321	Male	53	Kindergarten	27	46	Male	0.77	0.747	0.618	0	0.044

In brief, consistency and heterogeneity were confirmed in the symptom modalities identified in these participants. Therefore, an intervention protocol is recommended to amplify the remaining functions (e.g., language) and restore undeveloped attributes (e.g., sensory).

## Discussion

In this study, we aimed to establish a personalized recommendation algorithm for rehabilitation interventions in children and adolescents with ASD. Our study utilized ABC to obtain information to depict autistic symptoms for the following analysis. First, the attributes are defined in detail according to the DSM-5-TR. Second, a Q matrix was constructed to present the interaction among items and attributes. Finally, we conducted model selections to select the optimal CDM to build a personalized recommendation algorithm. Our results showed that GDINA is the most suitable model to produce a cognitive diagnosis or simulate the interactions among attributes and items. Twelve symptom modalities are recognized in our sample that depicts 12 dysfunction patterns based on ABC attributes. We proposed a recommendation diagram to simulate patient prognosis based on cluster analysis using symptom confirmation probability. We found that sensory and related functions should receive prior attention and that language function is identified as the most difficult target for ASD interventions. Numerous samples were randomly selected from our sample to illustrate the clinical practice in a real scenario. These findings serve as a preliminary test for more standardized research that applies cognitive diagnostic methods.

Whether the response pattern of children with ASD in ABC could display reasonable fitness to CDM?

In our study, choosing a reasonable model for simulating the response patterns of children and adolescents in ABC was the primary question. We used a relative index involving the AIC and BIC as the evaluation criteria (Vrieze, 2012). GDINA presented the best performance among all the CDMs in this study. This result confirms that the following analysis was conducted based on solid prior assumptions. To achieve more conservative parameters in our algorithm, BIC ensures that as we involve more samples in our study, the number of parameters in the true model is finite (Schauberger and Mair, 2020). This means that, as the sample grows large enough to represent the whole population, we will ultimately obtain the true model. On the contrary, AIC ensures that, even though we cannot recruit a representative sample in this population, the true model is still in the candidate model set (Vrieze, 2012). To our knowledge, our study is the first to apply CDM to simulate the response pattern in children and adolescents with ASD. Therefore, we cannot assure whether our sample size is reasonable to avoid possible conditions such as overfitting or non-fitting in model simulations. Hence, we chose GDINA with reasonable AIC and BIC values.

Could CDM reflect symptomological differences in children with different characteristics based on ABC components or attributes?

GDINA assumes that each behavior represented by each item should be observed in those who display related dysfunctions. The correlation analysis revealed that the ABC score was significantly related to the number of dysfunctions. This implies that the symptom patterns generated by GDINA using the ABC attributes are reasonable. Therefore, we found that the number of children and adolescents who display patterns “11111” is close to the number of children classified as having severe autism by CAR1. We also found that the number of children who display the pattern “00000” also approximates the number of children classified as non-autistic by CARS1. In line with previous findings, we confirmed heterogeneity in the ASD phenotype (Masi et al., 2017). We identified 28 symptom modalities in the study sample. Due to the unequal proportions of children with different education levels, these rare symptom modalities cannot be assigned to more patients.

Could CDM simulate progress in children with ASD according to symptomology characteristics in our sample?

We conducted a cross-sectional study to simulate the possible developmental diagrams of children and adolescents with ASD. Our results found that autistic symptoms did not develop in a uniform form but rather were characterized by periods of progress, particularly during the early stages. The irregular progression highlights the complexity of autistic symptoms, which is influenced by factors such as age, gender, and education (Peng et al., 2024a). These insights emphasize the importance of tailored interventions to alleviate autistic symptoms in children and adolescents with different symptom modalities. Physicians and therapists can design targeted interventions and scaffolded instructions to address different dysfunctions in symptom modalities.

How to construct a personalized intervention protocol according to the results of CDA?

Finally, the personalized analysis of students' mastery probabilities, as illustrated in our results, highlights the heterogeneity in individual strengths and weaknesses among children and adolescents, again addressing personalized interventions. For example, although ID1395 and ID1423 scored the same points on CARS1 and ABC, ID1395 can benefit from interventions that utilize his or her strength in language, and ID1423 can benefit from protocols that focus on sensory and related functions. In clinical practice, by leveraging data-driven insights, physicians and therapists can design adaptive learning pathways that continuously promote trial and error in protocol construction.

### Clinical applications

CDA and CDM have been widely used in educational research for their ability to enhance traditional evaluation methods by generating detailed diagnostic information. Previous studies have used CCT and IRT to demonstrate the clinical application of ABC in children and adolescents with ASD. However, these studies faced limitations in illustrating the interaction between dysfunctions and items, as well as limited instruction in designing personalized interventions. Hence, this study was conducted to address the above-mentioned gaps by constructing an attribute matrix using items and dysfunctions to explore more deeply the data that consists of ABC scores and related information. We have also proved the potential of CDA in understanding symptom modalities in children and adolescents with ASD. Detailed applications of personalized analysis are presented using randomly selected samples.

## Conclusion

Our study developed a personalized recommendation algorithm using CDA in designing individualized interventions for children and adolescents with ASD. Through an in-depth analysis of ABC data from our previous study, meaningful conclusions were drawn, providing insights into patients' symptom modalities. Moreover, comparisons among CDA results, ABC, and CARS1 scores ensured that CDA results are trustworthy in the following analysis. First, the CDA results confirmed the heterogeneity of the ASD symptoms. Importantly, the information derived from the CDA allowed for the construction of a possible development diagram of the functions defined by ABC. While these results are theoretically sound and reasonable, they remain data-driven findings. Further empirical validations, particularly through experience with rigorous design, are necessary to confirm the alignment between real-world practices and data-driven models.

## Data Availability

The datasets presented in this article are not readily available due to personal privacy. Requests to access the datasets should be directed to: 18096723g@connect.polyu.hk.
